# Appropriate Duration of Antimicrobial Treatment for Prosthetic Joint Infections: A Narrative Review

**DOI:** 10.3390/antibiotics13040293

**Published:** 2024-03-23

**Authors:** Jaime Lora-Tamayo, Mikel Mancheño-Losa, María Ángeles Meléndez-Carmona, Pilar Hernández-Jiménez, Natividad Benito, Oscar Murillo

**Affiliations:** 1 Department of Internal Medicine, Hospital Universitario 12 de Octubre, Instituto de Investigación Biomédica imas12 Hospital 12 de Octubre, Facultad de Medicina, Universidad Complutense de Madrid, 28041 Madrid, Spain; mikel.mancheno@gmail.com (M.M.-L.); pilihj@hotmail.com (P.H.-J.); 2 Spanish Group for the Study of Bone and Joint Infections, Spanish Society of Clinical Microbiology and Infectious Diseases (GEIO-SEIMC), 28003 Madrid, Spain; nbenito@santpau.cat (N.B.); omubio@gmail.com (O.M.); 3 CIBERINFEC—CIBER Enfermedades Infecciosas, Instituto de Salud Carlos III, 28029 Madrid, Spain; 4 Department of Microbiology, Hospital Universitario 12 de Octubre, Instituto de Investigación Biomédica imas12 Hospital 12 de Octubre, 28041 Madrid, Spain; marmelcar@gmail.com; 5 Infectious Diseases Unit, Hospital de la Santa Creu i Sant Pau, Institut d’Investigació Biomèdica Sant Pau (IIB SANT PAU), Universitat Autònoma de Barcelona, 08193 Barcelona, Spain; 6 UQ Centre for Clinical Research (UQCCR), The University of Queensland, Brisbane 4072, Australia; 7 Department of Infectious Diseases, Hospital Universitario Bellvitge, IDIBELL (Instituto de Investigación Biomédica de Bellvitge), 08908 L’Hospitalet de Llobregat, Spain

**Keywords:** biofilm, bone and joint infection, antimicrobial stewardship, arthroplasty infection, periprosthetic joint infection, implant-associated infection

## Abstract

Prosthetic joint infections are considered difficult to treat they needing aggressive surgery and long antimicrobial treatments. However, the exact duration of these therapies has been established empirically. In the last years, several studies have explored the possibility of reducing the length of treatment in this setting, with conflicting results. In this narrative review, we critically appraise the published evidence, considering the different surgical approaches (implant retention [DAIR] and one-step and two-step exchange procedures) separately. In patients managed with DAIR, usually treated for at least 12 weeks, a large, randomized trial failed to show that 6 weeks were non-inferior. However, another randomized clinical trial supports the use of 8 weeks, as long as the surgical conditions are favorable and antibiotics with good antibiofilm activity can be administered. In patients managed with a two-step exchange procedure, usually treated during 6 weeks, a randomized clinical trial showed the efficacy of a 4-week course of antimicrobials. Also, the use of local antibiotics may allow the use of even shorter treatments. Finally, in the case of one-step exchange procedures, there is a trend towards reducing the length of therapy, and the largest randomized clinical trial supports the use of 6 weeks of therapy.

## 1. Introduction

Foreign body-associated infections are difficult to treat. In these biofilm-associated infections, the bacteria express phenotypic tolerance to antibiotics and the immune system is ineffective [1]. Treatment often requires a surgical approach, commonly including removal of the foreign body [2].

In the specific case of prosthetic joint infection (PJI), the surgical treatment of reference is removal of the arthroplasty, usually followed by implantation of a new device in a one- or two-step exchange procedure [3]. As an alternative, carefully selected patients with acute PJI may benefit from a more conservative surgical strategy, in which thorough debridement, antibiotics, and implant retention (DAIR) may provide a reasonable chance of success [3,4,5]. Sometimes a curative surgical strategy is not possible, and chronic, antibiotic suppression treatment is used, theoretically indefinitely, with the aim of maintaining a functional and pain-free prosthetic joint [6].

Focusing on eradication strategies (prosthesis removal or DAIR), long-term antimicrobial treatment is usually recommended. The rationale for prolonged treatment is based on the difficulty of treating biofilm-embedded bacteria, the conventional treatment for osteomyelitis, and accumulated experience of PJI [3,7,8,9]. However, specific recommendations on the most appropriate duration of treatments are basically empirical [8], and tend to vary according to the surgical treatment and etiology of the infection. In this context, largely based on expert recommendations, the 2013 IDSA guidelines recommend 3 to 6 months for staphylococcal PJI managed with DAIR, but 4 to 6 weeks for other etiologies. The recommendations for patients managed with a one-step exchange procedure are similar, and a treatment duration of 4 to 6 weeks is suggested for those undergoing a two-step exchange [10].

However, there is a set duration of antimicrobial therapy beyond which there is no further increase in the success rate, while prolonging treatment only increases the risk of toxicity. In a retrospective study with more than a hundred episodes of PJI managed with DAIR, Byren et al. elegantly showed that patients treated for at least 6 months had the same likelihood of infection relapse after antimicrobials were stopped, regardless of treatment duration [11]. Thus, the need for such lengthy treatments should be weighed against their potential toxicity, as well as the ecological impact and the emergence of antimicrobial resistance [12,13,14,15].

In recent years there has been a trend towards shortening treatments, in line with other infections. In a bold move that went further than the official recommendations of medical societies, there was a strong consensus (>90% agreement) at the 2018 International Philadelphia Meeting on Musculoskeletal Infections for reducing the treatment duration for PJI managed with DAIR to 6 weeks [16], although the level of evidence to support this policy was weak. As we shall discuss below, a recent randomized controlled trial failed to prove that such a short treatment was as effective as longer therapies [17].

Several meta-analyses comparing short and long treatments for PJI have been published in recent years [18,19,20], all of which have consistently concluded that the available data favor the use of short treatment schedules; nevertheless, the results of the largest clinical trial performed to date contradict these results [17]. The meta-analyses included studies with PJIs in different sites, with different microbial etiologies and surgical management strategies, often using different definitions (of PJI and/or outcomes) and durations of antimicrobial treatment.

In this narrative review, we critically appraise the published evidence on the length of treatment for PJI, considering the main surgical approaches (DAIR, one-step and two-step exchange procedures) separately. We shall also try to identify a reasonable interval of time beyond which antimicrobials may be safely discontinued.

## 2. Search Strategy and Selection Criteria

For the search, the PubMed database was used, combining the terms ‘antibiotic length’, ‘antibiotic duration’, ‘antimicrobial duration’, ‘short course antibiotic’, ‘short course antimicrobial’, ‘short-term antibiotic’, ‘short-duration antibiotic’, ‘prolonged antibiotic’, ‘extended antibiotic’, ‘long-duration antibiotic’, ‘prosthetic joint infection’, ‘periprosthetic joint infection’, and ‘arthroplasty infection’. Abstracts were reviewed and papers addressing the influence of length of antimicrobial therapy on outcome were selected. References included in those articles were also consulted to review previous original studies. Excluded were studies of antimicrobial prophylaxis, chronic suppressive antimicrobial therapy, and fungal or mycobacterial PJI. For the purposes of this review, studies that addressed treatment duration without specifying the type of surgical management, and those that did not control for potential survivor bias were not included [21]. Studies of definite arthrodesis with no orthopedic hardware, which pose no risk for a new prosthesis, were also excluded from this review. We discarded papers written in languages other than English, French, or Spanish. Finally, we aimed to assess the efficacy of total duration of antimicrobial treatment in PJI, regardless of the route of administration (oral or intravenous). Other major studies in the field of osteoarticular infection and PJI have shown that, after a few days of intravenous treatment, oral treatments are a valid therapy [22,23].

## 3. Prosthetic Joint Infection Managed with DAIR

### 3.1. The Standard of Care: At Least Twelve Weeks of Treatment

The recommended duration of antibiotic treatment for patients managed with DAIR has changed over time, and most studies have focused on staphylococcal infection. Traditionally, and especially in North America, postoperative treatment has mainly been based on the administration of intravenous beta-lactams or glycopeptides for 4–6 weeks, frequently followed by long-term oral suppressive treatments [24,25,26,27,28]. In Europe, since the 1990s, highly bioavailable antibiotics with activity against biofilm-embedded bacteria (mainly rifampin and fluoroquinolones) have been successfully used for long but limited periods of several months (3 to 9 months) [29,30]. Finally, a randomized clinical trial published in 1998 laid the foundations for a treatment of 3 or 6 months for hip and knee staphylococcal PJI, respectively [31].

A number of attempts to reduce the minimum duration of 12 weeks have been explored over the last two decades, including some case series, four observational studies [32,33,34,35], one pre-post-study [36], and two randomized clinical trials [17,37] (Table 1). The common background to all these papers is frequent use of rifampin-based combinations for staphylococcal infections together with fluoroquinolones, when possible. Taken together, these studies provide evidence for the possibility of reducing the duration of treatment from 12 weeks to either 8 or 6 weeks.

### 3.2. Twelve Weeks versus Eight Weeks of Treatment

A number of observational studies without control group that used antibiotics for 8 to 12 weeks reported similar results to those previously published with longer treatments [38,39,40,41]. In addition, after changing the treatment duration policy in patients managed with DAIR, Puhto et al. published a pre-post study comparing reductions in treatment duration from 3 and 6 months for infected hip and knee prostheses, respectively, to 2 and 3 months. They found no differences patient outcomes in a per-protocol and intention-to-treat analysis (86% and 58% cure rates, respectively). Patients belonging to the short and long treatment groups were very similar, except for a higher rate of sinus tract and higher C-reactive protein values in the latter, which may account for a worse prognosis [36].

Of note, some studies by the Spanish REIPI Network also addressed the impact of treatment duration in PJI. In a large multicenter retrospective case series of staphylococcal PJI, no differences in relapse were observed between patients regardless of treatment duration, which ranged from 60 to more than 90 days. Only patients who had completed a planned course of antibiotics without failure were included in that analysis to avoid a survivor bias [34]. Tornero et al. reported similar results in 163 episodes of post-surgical PJI caused by a variety of microorganisms, treated mainly with rifampin-based combinations or fluoroquinolones [35]. Some other observational studies on risk factors for DAIR failure have noted a higher likelihood of relapse in PJI patients receiving shorter courses of antimicrobial therapy [42,43,44]. However, the results could be explained by a survivor bias, in which the reason for receiving the short treatment is the failure itself, and not the other way round, thus reversing the cause–consequence effect [21,45].

**Table 1:** Comparative studies assessing the efficacy of shorter treatments in infections managed by DAIR.

Reference	N ^1^ (Short/Long)	Design	Length of Therapy	Antibiotics	Etiology	Results (Cure Rates)
Bernard et al., 2010 [32]	60 (20/40)	Prospective observational, single-center, non-randomized	6 weeks vs. 12 weeks	Various, high use of rifampin and fluoroquinolones	Various (staphylococci ≈ 66%)	90% cure with the short treatment vs. 55% with the long treatment
Puhto et al., 2012 [36]	132 (72/60)	Retrospective observational, single-center, pre-post-design	2–3 months vs. 3–6 months ^2^	Rifampin-based combinations for GP and fluoroquinolones	Various	Non-inferiority of short treatments. Cure rates: ITT—Long 57%, Short 58% (*p* = 0.85) PP—Long 89%, Short 87% (*p* = 0.78)
Lora-T. et al., 2013 ^4^ [34]	231 (52/52/127)	Retrospective observational, multicenter	<61 days 61–90 days >90 days	Various (>75% rifampin-based combinations)	*Staphylococcus aureus*	<61 days—75% 60–90 days—77% >90 days—77% (*p* = 0.434)
Tornero et al. 2016 ^5^ [35]	143	Retrospective observational, single center	Variable	Various (including 88% rifampin-based for GP and 90% quinolones for GN)	Various	126 cases of no failure: 79 days of treatment (IQR 53–102) 17 cases of failure: 58 days of treatment (IQR 46–111) (*p* = 0.403) 6 cases of relapse: 79 days of treatment (IQR 48–145) (*p* = 0.942)
Lora-T. et al., 2016 [37]	63 (30/33)	Randomized, multicenter, open clinical trial	8 weeks vs. 3–6 months ^3^	Levofloxacin plus rifampin	Staphylococci	Trend towards non-inferiority. Cure rates: ITT—Long 58%, Short 73% (Δ = −15.7 95%CI −39.2% to +7.8%) PP—Long 95%, Short 92% (Δ = +3.3% 95%CI −11.7% to +18.3%)
Chaussade et al., 2017 [33]	87 (44/43)	Retrospective observational, multicenter	6 weeks vs. 12 weeks	Rifampin-based combinations for GP and fluoroquinolones	Various (staphylococci ≈ 40%)	Cure rates: 67.4% in the long treatment group 70.5% in the short treatment group (aOR 0.76, 95%CI 0.27–2.10)
Bernard et al., 2021 [17]	151 (75/76)	Randomized, multicenter, open clinical trial	6 weeks vs. 12 weeks	Various, including the use of rifampin and fluoroquinolones	Various (*S. aureus* ≈ 30–40%)	Failure rate for 6 weeks: 30.7% Falure rate for 12 weeks: 14.5% Difference: 16.2% (95%CI: 2.9% to 29.5%)

DAIR—debridement, antibiotics, and implant retention. GP: Gram-positive microorganisms. ITT: intention-to-treat analysis. PP: per-protocol analysis. 95% CI: 95% confidence interval. ^1^ Refers to the number of patients managed with DAIR (number of patients treated with a short treatment/number of patients treated with a long treatment). ^2^ Long schedule consisted of 3 months for hip prostheses and 6 months for knee prostheses, and short treatments consisted of 2 and 3 months for hip and knee prostheses, respectively. ^3^ Long schedule consisted of 3 months for hip prostheses and 6 months for knee prostheses. ^4^ Multicenter cohort including 345 cases of staphylococcal PJI managed with DAIR; this analysis was performed on patients who had finished a scheduled treatment with no signs of failure. ^5^ Patients with a postoperative infection (maximum of 90 days after index surgery) undergoing DAIR within the first 21 days of symptoms; only patients with no failure during treatment and a minimal 2-year follow-up were included.

Finally, a Spanish randomized clinical trial addressed the non-inferiority of a short course (8 weeks) of levofloxacin plus rifampin to longer regimens of the same antimicrobial combination (3 months for hip prostheses and 6 months for knee prostheses) [37]. The study was conducted under a non-inferiority hypothesis, with a maximum Δ value of 15% in favor of long treatments. The included patients had acute infections caused by staphylococci (either coagulase-negative or *S. aureus*) and met the commonly accepted eligibility criteria for DAIR [3]. The study was underpowered due to the small number of patients finally recruited (*n* = 63), which also resulted in less than perfectly homogeneous groups (there was a higher percentage of polymicrobial infection among patients randomized in the long-schedule group). Despite these limitations, the non-inferiority hypothesis was proven in the intention-to-treat analysis, where the rates of success in the long- and short-treatment arms were 56.6% and 73.3%, respectively (Δ = −15.7%, 95% confidence interval [95%CI] −39.2% to +7.8%). In a per-protocol analysis, cure rates were 95.0% and 91.7%, but the non-inferiority hypothesis was not proven (Δ = +3.3%, 95%CI −11.7% to +18.3%). Based on these studies, the American Academy of Orthopaedic Surgeons acknowledged the possible efficacy of an 8-week schedule [46].

### 3.3. Twelve Weeks versus Six Weeks of Treatment

A French research group published two observational studies with similar clinical results. In a prospective observational single-center study, Bernard et al. compared a large number of patients who received either 6 or 12 weeks of antimicrobial therapy (at the discretion of the treating physicians), 60 of whom were managed with DAIR (20 for 6 weeks and 40 for 12 weeks). Only two patients failed in the short-term group (90% cure rate) [32]. The same research group published a new retrospective analysis involving three hospitals and a higher number of patients and again, found no differences between 6 and 12 weeks of treatment (70% and 67% cure rates, respectively) [33].

Finally, Bernard et al. recently published the DATIPO study [17], a French multicenter randomized open-label clinical trial that included a large sample of patients with PJI (*n* = 404), managed with various surgical and antimicrobial treatments. Based on their previous studies, Bernard et al. hypothesized that 6 weeks of treatment would be non-inferior to 12 weeks, with a Δ value of 10%. The majority of patients were treated with DAIR (*n* = 151) and in this group the rate of failure was significantly higher among those treated for only 6 weeks (31% vs. 15%; risk difference 16.2% [95% confidence interval 2.9% to 29.5%]).

In summary, a number of observational studies and one clinical trial with a small sample size suggest that treatments of less than 12 weeks (and aiming for 8 weeks) could be successfully used for DAIR, while a recent large clinical trial failed to prove that 6 weeks of treatment was enough. Therefore, while we can conclude that the duration of antimicrobial therapy in patients treated with DAIR should be longer than 6 weeks, 3 to 6 months of therapy is probably not necessary. The optimal duration of therapy for these patients could be somewhere between these two.

## 4. Prosthetic Joint Infection Managed with a Two-Step Exchange Procedure

The normal total duration of systemic antibiotics for PJI managed with a two-step exchange procedure is 4 to 6 weeks (IV, or oral plus IV) [10]. However, some studies have suggested that this interval could be significantly shortened. A two-stage exchange of the prosthesis has two major advantages for the management of PJI. First, removal of the foreign body and accompanying biofilm greatly simplifies treatment, as with many other device-associated infections [2]. Second, a cement spacer is often used to preserve the joint space and ease reimplantation of the prosthesis. These spacers can be loaded with antibiotics that deliver high concentrations of antimicrobials to the surgical site, which could not be reached by other means [3,5]. Most experience with local antibiotics relies on vancomycin and aminoglycosides (mainly gentamycin, but also tobramycin) [47].

In a randomized trial including patients undergoing a two-step exchange procedure, Nelson et al. observed a 15% failure with local antibiotics alone (without systemic antimicrobials) compared to 30% with systemic antibiotics alone (and no local antimicrobials) [48]. Similarly, in another randomized trial, Berwanger et al. found that the success rate was higher in patients managed with local plus systemic antibiotics than in those receiving systemic antimicrobials alone [49]. Indeed, current recommendations advocate combining local and systemic antimicrobial strategies in the setting of a two-step exchange procedure for PJI [3,5,10].

### 4.1. Local Antimicrobials and Shorter Treatments for PJI

In the first decade of this century, various prospective observational non-comparative analyses by two specialist orthopedic centers in northern England reported high rates of success based on local antimicrobial therapy and a very short course of systemic antibiotics (24 h to 14 days) (Table 2). Although these studies were non-comparative, the number of patients who required additional debridements before reimplantation, the rate of positive intraoperative cultures at the second-stage surgery, and the proportion of persistent or relapsing infections were comparable to previous series [50,51,52,53,54].

Four comparative studies provided additional proof of the importance of local antimicrobials. The analyses published by Mittal et al. and El Helou et al. showed that it was possible to reduce the duration of therapy to less than 6 weeks and 4 weeks, respectively [55,56]. In 2009, Hsieh et al. reported a similar rate of success for patients treated for 4 to 6 weeks and those treated for 7 days after prosthesis removal [57]. Ma et al. recently reported similar results [58].

### 4.2. Shorter Treatments Independent of Local Antimicrobials

In 2019, Benkabouche et al. provided evidence of the successful shortening of systemic antimicrobials after implant removal, independent of local antibiotics. In an open randomized controlled trial, they proved that a 4-week schedule of treatment was non-inferior to 6 weeks of antibiotics in a cohort of 123 patients with bone and joint infection whose orthopedic hardware had been removed. Among these patients, there were 39 episodes of PJI treated with a two-step exchange procedure. Of particular interest, only two patients (5%) were receiving local antibiotics (tobramycin) [59].

**Table 2:** Studies assessing the efficacy of short treatments in prosthetic joint infection managed with implant removal.

Ref.	N/Location	Study Design	Etiology	Local ATB	Duration of Systemic ATB	Follow Up (Months)	Outcome		
							Additional Debridements	PIOC at Reimplanttion	Relapse/ Persistence
Taggart et al., 2003 † [53]	33/Hip & Knee	Prospective observational, single-center, non-comparative	93% Gram-positives 71% staphylococci	Vancomycin	5 days	67	0%	9%	3%
Hoad-Reddick et al., 2005 † [51]	52/Knee	Prospective observational, single-center, non-comparative	Various (63% staphylococci)	Various ^1^	24 h	56	12%	16%	9%
Hart & Jones, 2006 ‡ [50]	48/Knee	Prospective observational, single-center, non-comparative	96% Gram-positives 76% staphylococci	Vancomycin + Gentamycin	14 days	49	13%	23%	13%
Stockley et al., 2008 † [52]	114/Hip	Prospective observational, single-center, non-comparative	Various (61% staphylococci)	Various ^1^	24 h	74	4%	16%	12%
Whittaker et al., 2009 ‡ [54]	44/Hip	Prospective observational, single-center, non-comparative	All Gram-positives (72% staphylococci)	Vancomycin + Gentamycin	14 days	49	7%	2%	7%
McKenna et al., 2009 [60]	31/Hip	Retrospective, observational, single-center, non-comparative	All Gram-positives (77% staphylococci)	Various ^1^	5 days	35	0%	0%	0%
Mittal et al., 2007 [55]	37/Knee	Retrospective, observational, multicenter, comparative	Methicillin-resistant staphylococci	Various, in 95% of patients	≥6 weeks iv vs. <6 weeks iv	51	-	0%	Short: 2/15 (13%) Long: 2/22: 9% (*p* = 0.07)
Hsieh et al., 2009 [57]	99/Hip	Retrospective, observational, single-center, comparative ^3^	67% Gram-positives 53% staphylococci	Various	4–6 weeks ^2^ vs. 7 days	43	Long 2/46 (4%) Short 1/53 (2%)	-	Long: 2/46 (4%) Short: 3/53 (6%)
El Helou et al., 2011 [56]	208/Hip & Knee	Retrospective, observational, single-center, comparative, propensity score-adjusted	Mainly Gram-positives. 62% staphylococci	Vancomycin ± Tobramycin	4 weeks ± 7 d vs. 6 weeks ± 7 d	60	-	Short: 6.1% Long: 8.7%	Short: 16% Long: 27%
Benkabouche et al., 2019 [59]	39 ^4^/Hip & Knee	Single-center, open, randomized clinical trial	Various	Only 2 cases (5%); tobramycin	6 weeks vs. 4 weeks	26	No significant differences were observed in the whole study and the PJI group		
Ma et al, 2020 [58]	64/Knee	Retrospective, observational, single-center, comparative	Various (69% staphylococci)	Vancomycin (± aminoglycosides)	4–6 weeks vs. ≤7 days	75	Need for salvage antimicrobials or surgery Long: 11/43 (26%); Short: 3/21 (14%)		
Bernard et al., 2021 [17]	81/Hip & knee	Multicenter, open, randomized clinical trial	Various (≈40% *S. aureus*)	Unknown	6 weeks vs. 12 weeks	≥24	Failure: 6 w: 6/40 (15%); 12 w: 2/41 (5%) (*p* > 0.05) Difference: 10.1% (95%CI −0.9–22.2), favoring long treatments		

The middle horizontal line segregates non-comparative (above) from comparative (below) studies. ATBs: antibiotics. PIOCs: positive intraoperative cultures. ^1^ Additional antibiotics were added according to preoperative cultures, but in most cases, vancomycin plus aminoglycosides were used. ^2^ In the long treatment arm, 4 weeks of intravenous antibiotics were prescribed. Additionally, 2 supplementary weeks of oral antimicrobials could be administered, provided there were oral options available. ^3^ Pre-post-comparative design. ^4^ Inclusion of 123 cases of bone and joint infection where all orthopedic hardware had been removed and no immediate osteosynthesis or prosthesis implantation had been performed. Of these, there were 39 cases of prosthetic joint infection managed with prosthesis removal. † From the Sheffield Teaching Hospitals Trust (Sheffield, England). ‡ From the Robert Jones and Agnes Hunt Orthopaedic Hospital NHS Trust (Oswestry, England).

A supplementary issue affecting patients undergoing a two-step exchange procedure that has recently generated considerable debate is antimicrobial treatment after reimplantation of the new arthroplasty. No firm recommendations can be given at this point, as many authors consider the cure of the infection to be a given if cultures taken during the second stage of surgery are negative (after withholding antibiotics for at least 2 weeks) [3,5,10]. Two observational PJI studies [61,62] and a randomized controlled trial [63] have shown that a 3-month course of oral antibiotics after reimplantation of the prosthesis is associated with a lower likelihood of relapse. At the same time, however, the vast majority of new episodes of infection were not caused by the same microorganism, suggesting that prolongation of antibiotics after the second stage, rather than treatment of the original infection, may actually be extended antimicrobial prophylaxis (or pre-emptive treatment) in a subset of patients with a high likelihood of developing a new episode of PJI [64].

### 4.3. Large Clinical Trials

In contrast to the studies suggesting that shorter treatments may be valid, the previously mentioned DATIPO trial failed to prove the non-inferiority of 6 weeks of treatment to a 12-week regimen in the context of a two-step exchange procedure [17]. It remains paradoxical that these patients were actually randomized to receive 6 versus 12 weeks of treatment, though. This was a secondary analysis, which may have been underpowered due to the sample size of the subgroup. Differences in rates of failure between the two treatment durations were not in fact statistically significant (15.6% for the short-treatment group, 4.9% for the long-treatment group 6, Δ = 7.9% (95%CI −0.2–16.0%).

At the time of writing, an ambitious and promising multicenter randomized controlled trial is underway in England. The SOLARIO trial is evaluating whether a 7-day course of antibiotics would be enough to treat bone and joint infections (including PJI) managed with hardware removal and local antibiotics, as compared with the usual longer courses of antibiotics [65].

In summary, in patients undergoing a two-step exchange procedure, there is the potential advantage of two routes of antibiotic administration. Regardless of local antimicrobials, there is some evidence to suggest that four weeks could be as effective as six weeks. Furthermore, the use of local active antibiotics has shown a greater promise for reducing the duration of systemic antimicrobials to just a few days. Nevertheless, a subanalysis of the DATIPO study, the most important randomized trial to date, calls these results into question. We look forward therefore to the results of the ongoing SOLARIO trial [65].

## 5. Prosthetic Joint Infection Managed with a Single-Step Exchange Procedure

Spurred on by the advantages of resolving chronic infection with just one operation, the number of case series reporting the results of single-step exchange procedures has increased over time, both for hip and knee prostheses [66,67]. The use of local antimicrobials mixed in bone cement was common in most of these series [68,69,70,71,72,73,74,75,76,77,78,79,80,81,82,83,84,85,86,87,88,89,90,91,92,93,94,95,96,97,98,99,100,101,102,103].

**Figure 1:**
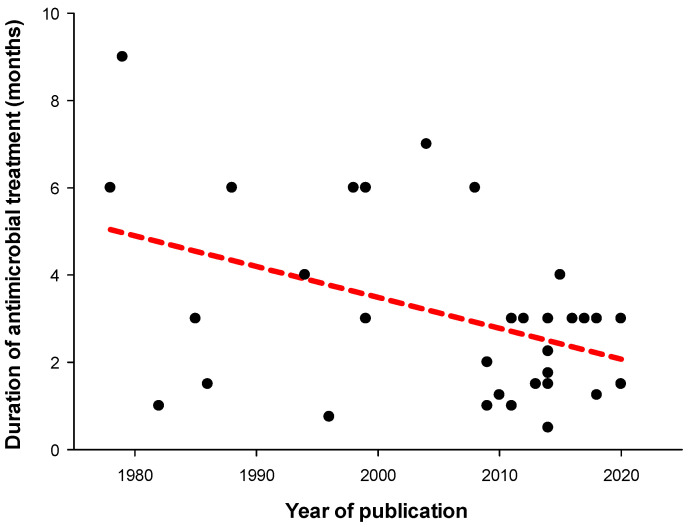
Published duration of systemic antimicrobial therapy (intravenous plus oral treatments) over time in cases of prosthetic joint infection managed with a single-step exchange procedure [68,69,70,71,72,73,74,75,76,77,78,79,80,81,82,83,84,85,86,87,88,89,90,91,92,93,94,95,96,97,98,99,100,101,102,103]. A regression line (doted red line) depicts a linear regression the data.

There is a tendency to shorten treatments over time (Figure 1) [68,69,70,71,72,73,74,75,76,77,78,79,80,81,82,83,84,85,86,87,88,89,90,91,92,93,94,95,96,97,98,99,100,101,102,103]. Case series published before and after 2005 report a median treatment duration of 5 months (IQR 1.9–6.0) and 2.6 months (IQR 1.5–3) (*p* = 0.029), respectively [68,69,70,71,72,73,74,75,76,77,78,79,80,81,82,83,84,85,86,87,88,89,90,91,92,93,94,95,96,97,98,99,100,101,102,103]. Notably, the success rate was not observed to decrease over time [67]. Chieffo et al. recently published a 90% success rate in 50 patients with PJI managed with single-stage revision and 6 weeks of antimicrobial treatment [104]. Still, patients undergoing a single-step revision have usually a more favorable clinical picture than those submitted to DAIR or to a two-stage exchange, therefore we cannot rule out that the good results observed are not influenced by a selection bias.

Finally, in the DATIPO study, the subset of patients managed with a single-step exchange procedure was the only subgroup in which 6 weeks of treatment was non-inferior to 12 weeks (*n* = 146; failure rates 4.0 and 2.8%, respectively; difference 1.2% [95%CI −4.8 to 7.1]) [17].

In summary, in patients undergoing a one-step exchange procedure for PJI, there is an empirical trend towards shortening the duration of antimicrobial treatment from 3–6 months to less than 3 months. Recent evidence supports the use of an even shorter course of 6 weeks.

## 6. Discussion and Future Directions

The field of infectious diseases is haunted by the emergence of multidrug-resistant microorganisms and the dearth of new effective treatments. Among other measures, antimicrobial stewardship programs have emphasized the need to adjust treatment durations and reduce exposure to antibiotics [105]. Increasing clinical evidence supports the use of short treatments for pneumonia, urinary infections, and bacteremia, among others [106]. In general, finding ways to shorten antimicrobial therapies has become a sign of the times at the beginning of the twenty-first century.

This trend extends to the field of bone and joint infection [107], including PJI. The aggressive management required to treat these infections refers not only to the surgery, but also to the administration of high doses of antimicrobials for long periods. Recommendations on the appropriate duration of treatments have changed over time, based on empirical results and especially on the route of administration used. When administered intravenously, antibiotics are given for 4 to 6 weeks, following the standard recommendations for osteomyelitis [7] and imitating other difficult-to-treat infections such as infective endocarditis. Extending intravenous antibiotics beyond 6 weeks requires more complex infrastructure (e.g., OPAT) and is not without adverse events [108]. The end of the twentieth century witnessed the emergence of effective and highly bioavailable oral antibiotics, namely rifampin and fluoroquinolones, which are administered for longer periods. In the particularly difficult setting of DAIR, the use of these antibiotics in well-selected patients has led to high rates of success.

However, there is still a gray area between recommending shorter intravenous treatments (4–6 weeks) and longer oral treatments (3–6 months) for the same infection, especially when using antimicrobials with good bioavailability and antibiofilm profile [10]. Furthermore, the OVIVA (Oral versus Intravenous Treatment) trial showed that oral treatments were non-inferior to intravenous antibiotic therapy and expanded the gray area to a wide range of antimicrobial families [22]. A recently published Australian clinical trial also showed that oral treatments for PJI were non-inferior to the IV route [23].

The administration of antibiotics for long periods of time is not without the possibility of toxicity and ecological impact. In some instances, patients must undergo several surgeries, and are thus potentially exposed to superinfection with microorganisms resistant to previous antimicrobial therapies. Indeed, the dreaded PJI caused by *Candida* spp typically occurs in patients undergoing multiple surgeries exposed to various prolonged antimicrobial treatments [109].

In this review, we have looked at studies assessing the efficacy of shorter therapies for PJI. Tools to personalize treatment duration for each patient, such as C-reactive protein or other inflammatory markers, would be desirable, but these have proven to be of little use for predicting relapse [110,111]. Instead, we have focused on reports assessing clinical outcomes as a function of different treatment durations. The studies analyzed here are heterogeneous. Some of them address very specific clinical problems (such as the same type of PJI, etiology, surgical management, and antimicrobial treatment), while others include different types of infection, surgeries, microorganisms, and antimicrobial therapies. Bearing in mind these limitations, there is overall a significant body of literature to support the use of shorter courses of antimicrobials for these patients. Recently published meta-analyses, including many of the studies discussed here, showed no significant differences in outcome between short and long courses of antibiotics [18,19,20].

In this context, the failure of the recent DATIPO trial to prove that 6 weeks of treatment was non-inferior to 12 weeks has dampened the enthusiasm for shortening treatments [17]. However, as the authors of the manuscript acknowledge, the mixture of patients, etiologies, antibiotics, and especially surgical treatments, may have undermined the objective of the study. Analyzing length of therapy for both DAIR and two-step exchange may be as misleading as trying to determine the exact duration of any staphylococcal infection, regardless of whether it is a skin infection or infective endocarditis. Indeed, while it makes sense to compare 6 vs. 12 weeks for patients managed with DAIR, it remains counter-intuitive to do so in patients undergoing a two-step exchange, when a wealth of experience supports a maximum of 6 weeks.

In general therefore, while patients undergoing DAIR are likely to require more than 6 weeks of treatment, there is still a significant difference between 6 and 12 weeks; in this context, eight weeks may be sufficient to treat most infections [37,46]. However, some observations need to be made before blithely embarking on these short therapies. First, treatment with DAIR should be performed according to Zimmerli’s algorithm (short duration of symptoms, acute infections, good skin and soft tissue status, and stable implant) [3]. Second, surgical treatment should be thorough and complete, ideally performed by experienced, skilled surgeons, and including the exchange of removable prosthetic components (i.e., polyethylene liner) [34,112,113]. Third, it is not just the duration of antibiotics that is important, but also the choice: the antimicrobials used must have good activity against biofilm-embedded bacteria [35,114]. For staphylococcal infections, clinical results are better when treated with a rifampin-based combination, ideally with a fluoroquinolone [115]. In the case of Gram-negative PJIs, fluoroquinolones are also the treatment of choice [5,116,117]. If these antibiotics cannot be used either because of toxicity, allergy, or resistance, the success of a short course of treatment may be not guaranteed. This underscores the importance of an appropriate microbiological diagnosis in this difficult clinical setting. In cases where all the above conditions cannot be met, antimicrobials may need to be administered for longer periods.

In patients with PJI managed with a two-step exchange procedure, the use of local antibiotics seems to admit very short treatments, possibly no longer than 2 weeks. However, we still need larger comparative trials to be certain, and so we are still awaiting the results of the SOLARIO study [65]. In the meantime, we have a randomized clinical trial in which no local antibiotics were used that supports the use of 4 weeks of systemic treatment [59]. However, patients with PJI constituted a small subgroup of that trial, and confirmatory studies with more patients and involving more centers would be welcome.

Treatment with a one-step exchange procedure is becoming increasingly common. There is a lack of consistent evidence to suggest shorter treatments, although there is a definite trend towards reducing the length of therapies to as little as 6 weeks of treatment with no apparent impact on reported success rates [67]. It may be that the antibiotic-laden cement used in most of these case series is of help in treating the residual periprosthetic osteomyelitis after prosthesis removal.

Given the personal and economic costs of PJI, along with the catastrophic implications of a relapse, some authors would recommend indefinite chronic suppressive antimicrobial therapy both for DAIR and the prosthesis exchange strategy [10]. This may be a prudent and valid measure in particular cases, such as elderly patients with a high likelihood of relapse due to the nature of the infecting microorganisms or for other reasons, but it cannot be applied to all patients. It has been shown that PJI can be cured with appropriate surgical and medical treatment, and in this article we have aimed to review the possibility of shortening treatments for these patients.

In conclusion, there is increasing evidence to support the use of short treatments for patients with PJI managed with DAIR. Eight weeks of treatment is probably sufficient for most patients, as long as the conditions of treatment relating to thoroughness and the indication for surgery, and the appropriateness of antimicrobial therapy are met. For cases managed with a two-step exchange procedure, the use of local antibiotics probably allows the use of very short courses of treatment (1–2 weeks), but further well-designed clinical trials are needed to confirm these results. Less evidence is available on the duration of antimicrobial treatment after a one-step prosthesis exchange, but 6 weeks may be sufficient under favorable circumstances.
